# Metabolic Phenotypes and Step by Step Evolution of Type 2 Diabetes: A New Paradigm

**DOI:** 10.3390/biomedicines9070800

**Published:** 2021-07-09

**Authors:** Isabella D. Cooper, Kenneth H. Brookler, Yvoni Kyriakidou, Bradley T. Elliott, Catherine A. P. Crofts

**Affiliations:** 1 Translational Physiology Research Group, School of Life Sciences, University of Westminster, 115 New Cavendish Street, London W1W 6UW, UK; y.kyriakidou@westminster.ac.uk (Y.K.); b.elliott@westminster.ac.uk (B.T.E.); 2 Research Collaborator, Aerospace Medicine and Vestibular Research Laboratory, Mayo Clinic, Scottsdale, AZ 85259, USA; brookler.kenneth@mayo.edu; 3 Faculty of Health and Environmental Sciences, School of Public Health and Interdisciplinary Studies, Auckland University of Technology, Auckland 0627, New Zealand; catherine.crofts@aut.ac.nz

**Keywords:** hyperinsulinaemia, insulin resistance, osteocalcin, beta-hydroxybutyrate, phenotype, stages, serotonin, glucagon-like peptide-1, glucagon, type 2 diabetes, hyperglycaemia

## Abstract

Unlike bolus insulin secretion mechanisms, basal insulin secretion is poorly understood. It is essential to elucidate these mechanisms in non-hyperinsulinaemia healthy persons. This establishes a baseline for investigation into pathologies where these processes are dysregulated, such as in type 2 diabetes (T2DM), cardiovascular disease (CVD), certain cancers and dementias. Chronic hyperinsulinaemia enforces glucose fueling, depleting the NAD+ dependent antioxidant activity that increases mitochondrial reactive oxygen species (mtROS). Consequently, beta-cell mitochondria increase uncoupling protein expression, which decreases the mitochondrial ATP surge generation capacity, impairing bolus mediated insulin exocytosis. Excessive ROS increases the Drp1:Mfn2 ratio, increasing mitochondrial fission, which increases mtROS; endoplasmic reticulum-stress and impaired calcium homeostasis ensues. Healthy individuals in habitual ketosis have significantly lower glucagon and insulin levels than T2DM individuals. As beta-hydroxybutyrate rises, hepatic gluconeogenesis and glycogenolysis supply extra-hepatic glucose needs, and osteocalcin synthesis/release increases. We propose insulin’s primary role is regulating beta-hydroxybutyrate synthesis, while the role of bone regulates glucose uptake sensitivity via osteocalcin. Osteocalcin regulates the alpha-cell glucagon secretory profile via glucagon-like peptide-1 and serotonin, and beta-hydroxybutyrate synthesis via regulating basal insulin levels. Establishing metabolic phenotypes aids in resolving basal insulin secretion regulation, enabling elucidation of the pathological changes that occur and progress into chronic diseases associated with ageing.

## 1. Introduction

The hormone insulin is synthesized and secreted by pancreatic beta cells [1,2]. The commonly accepted principle is that insulin is secreted in a basal/bolus pattern, with the latter predominately released upon rising blood glucose (most likely from a meal) stimulus [3,4]. The mechanism by which bolus insulin is secreted from the pancreas is well established [5]; however, little is known about the mechanisms by which basal insulin is secreted.

Once we understand the processes by which insulin is secreted in both the basal and bolus states in a healthy person, we can begin to unravel the pathologies whereby these processes are dysregulated, such as in type 2 diabetes mellitus (T2DM), cardiovascular disease (CVD), certain cancers and dementias [6].

## 2. Bolus Insulin Secretion

The commonly accepted premise is that the primary function of insulin secretion is to regulate glucose uptake into muscle cells [7,8]. To better understand basal versus bolus insulin secretion regulation and physiological roles, a firm understanding of how glucose drives rapid insulin exocytosis is warranted.

**Figure 1:**
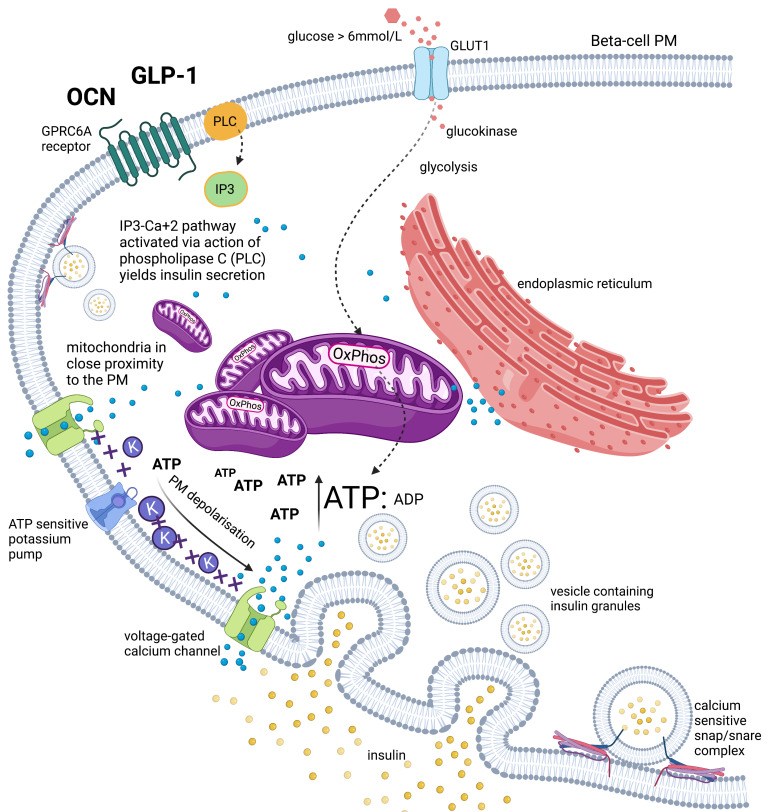
Schematic representation of beta-cell intracellular mechanisms involved in insulin secretion. Adenosine diphosphate (ADP), adenosine triphosphate (ATP), calcium (Ca^2+^), glucagon-like peptide-1 (GLP-1), glucose transporter 1 (GLUT1), G-protein coupled receptor 6A (GPRC6A), inositol-1,4,5-trisphosphate (IP3), plasma membrane (PM), osteocalcin (OCN), oxidative phosphorylation (OxPhos), phospholipase C (PLC), potassium (K^+^).

Glycaemic elevations, such as following an oral carbohydrate bolus, results in rapid entry of glucose into the pancreatic beta cells via GLUT1 and/or GLUT3 glucose transporters (Km = 6 mmol/L and Km = 1–1.4 mmol/L, respectively) (Figure 1) [9,10,11]. GLUT1 is the predominant glucose transporter in humans, its Km of 6 mmol/L indicates that this transporter is only activated when significantly high levels (above the physiological concentration of 5 mmol/L) of glucose are detected in the blood stream [3]. However, the high glucose affinity of GLUT3, suggests its role for metabolic fuel homeostasis during fasting periods where there would be glucose/carbohydrate deprivation/restriction. Theoretically, in this setting the GLUT3 receptor expression is upregulated.

Upon entry into beta cells, glucose is phosphorylated to glucose-6-phosphate by glucokinase (GK), an isozyme of hexokinase [12]. GK has a high Km value = 10 mmol/L, indicating a low affinity for glucose [13,14]. GK is predominantly only expressed in hepatocytes and pancreatic beta cells [13,15,16], and also found to be expressed by cells in the hypothalamus [17]. Glucose is catabolised through the glycolytic pathway to pyruvate, in the process generating reducing equivalents NADH. These enter the mitochondria to undergo a series of redox reactions to yield adenosine triphosphate (ATP) via the electron transport chain (ETC) oxidative phosphorylation (OxPhos) machinery coupled to ATPase. The ETC-OxPhos complexes are situated on the cristae of the inner mitochondrial membrane (IMM) [3]. Beta cells express low levels of lactate dehydrogenase, indicating a “preference” to fully oxidise glucose via OxPhos generating maximal ATP (~36 ATP/glucose molecule), as opposed to via the fermentation pathway (~2 ATP/glucose molecule) [18,19].

As glucose is fully oxidised through OxPhos, a surge of ATP is generated via the mitochondrial high electrochemical-potential gradient inner membrane (ΔΨm) [20]. As a result, there is a steep increase in the ATP concentration relative to adenosine diphosphate (ADP) [21]. The mitochondria are situated close to the beta cell plasma membrane (PM) [22]. The surge in ATP affects ATP-sensitive potassium channels (K_ATP_), causing them to close [23,24]. This depolarises the PM, causing PM voltage gated calcium channels (VGCC) to open [23,25]. The subsequent rapid influx of calcium divalent cations, through P/Q-type Ca^2+^ channels [26], activates the calcium sensitive SNAP/SNARE complexes that hold vesicles containing insulin granules in a “ready” docking position on the cytosolic side of the PM [27]. This induces the rapid exocytosis of insulin, which is referred to as the first phase response [28]. Further insulin is stored in “reserve pool” vesicles and released only in response to fuel secretagogues [27], known as the “second-phase”. This second phase of insulin release has an observed delay and insulin nadir after the first phase, and has a lower amplitude and longer direction, but only lasts while the beta cells are stimulated [20].

A whole host of aspects of the glucose-mediated insulin first phase response needs to be closely coordinated in-order to promote exocytosis of a high concentration of insulin granules, outside of the beta-cells basal pulsatile release. These include the expression of GK, nicotinamide adenine dinucleotide (NAD+) availability, and maintaining a high capacity to perform OxPhos that requires a high ΔΨm, in-order to generate the necessary surge in ATP concentration to affect the (K_ATP_) channels. Finally, calcium homeostasis must also be well regulated, as a PM concentration gradient and consequent signal:response ratio are required to elicit the exocytosis response. Therefore, calcium relocation mechanisms must also be effective [29].

## 3. Basal Insulin

In contrast to the above-described regulation of bolus insulin secretion, the regulation of insulin secretion in the basal phase is not well understood. It could be hypothesised that the same mechanism is used for insulin secretion in the basal state. It is recognised that GLUT3 has a lower Km for glucose (Km = 1–1.4 mmol/L) and in the fasted state GLUT3 may become upregulated, thereby increasing the role of glucose in basal insulin secretion regulation. However, if this were the case, increased insulin release would down regulate beta-hydroxybutyrate (BHB) synthesis. This is not corroborated by the observation of individuals in the fasted state, where a lower basal state of insulin and glucagon, with the presence of BHB, has been observed in people in habitual ketosis [30,31,32]. Another argument for it not being GLUT3 dominant stimulated in the basal state, is that glucose in the fasted state is at a relatively steady state with low degrees of magnitude in the blood, which, therefore, would not correspond to basal insulin oscillatory patterns.

**Figure 2:**
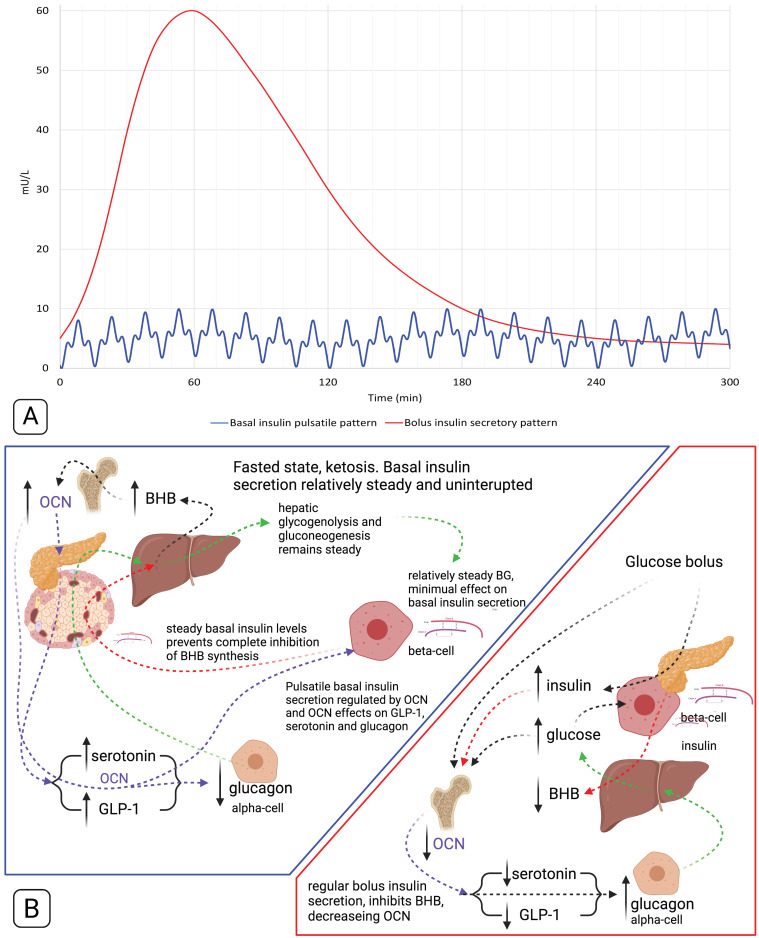
Schematic representations of basal and bolus insulin secretory patterns (**A**) and secretion regulation (**B**). (**A**) The red line conceptually models glucose bolus mediated insulin secretory response pattern (Kraft I) [36], and the blue line conceptually models basal insulin pulsatile secretory pattern, in metabolically healthy individuals [34]. (**B**) Schematic representation of the regulatory cycles of basal and bolus insulin secretion in metabolically healthy, habitually fasted individuals. Beta-hydroxybutyrate (BHB), blood glucose (BG), glucagon-like peptide-1 (GLP-1), osteocalcin (OCN).

Basal insulin is recognised to be released from pancreatic beta cells in a pulsatile rhythmic pattern, approximately every 4 min, in addition to circadian and ultradian periodicities (Figure 2A) [20]. Five-to-fifteen-minute fast oscillations modulate the ultradian periodicity, which has a range of 40 to 180 min [7,20,33]. It is believed that the modulatory fast oscillations are influenced by the degree of insulin resistance (IR) within an individual [34]. The bone derived hormone osteocalcin (OCN) activates beta cell calcium signalling via the GPRC6A receptor (Figure 1) and we hypothesise it is the OCN that regulates the oscillatory insulin secretion pattern (Figure 2B) [35].

The pulsatility pattern likely reduces the risk of negative consequences from potentially downregulating insulin receptors (INSR), which would result in further insulin resistance. Therefore, a pulsatile secretory pattern plausibly is more effective in regulating blood glucose levels [7]. In addition to contributing to sympathovagal balance, there is a tight coupling between the pancreatic ultradian periodicity and the neuroendocrine, cardiovascular and autonomic nervous systems [37].

Additional challenges in the understanding of pancreatic beta cell insulin secretion, include changes to the IMM. Mitochondria are the largest intracellular source of reactive oxygen species (ROS) [38,39,40], specifically from the activities of the ETC, as electrons “leak” and react with oxygen, forming superoxide [41,42]. Cellular mechanisms to counter ROS are facilitated by anti-oxidant enzymes such as mitochondrial superoxide dismutase (SOD2) and reduced glutathione (GSH) [43]. Both SOD2 and GSH require NAD+ [44]. Glucose oxidation has a greater NAD+ depletion effect over beta-oxidation or ketolysis; as a result, this increases ROS levels via a reduced ability to counter ROS [43,45,46,47]. Furthermore, excessive insulin signalling increases ROS levels via ceramide synthesis [48], which, in turn, leads to cellular apoptosis. Without sufficient anti-oxidative enzymes to manage the excessive ROS levels, the mitochondrial strategy is to increase the expression of uncoupling proteins (UCP2) in the IMM [20,49]. This causes the uncoupling of proton flow from the higher concentration within the inner membrane space of the mitochondrial double membrane lipid bilayer, into the mitochondrial matrix, without generating ATP. This results in a less hyperpolarised IMM, and thus resultant inability to generate the surge in ATP, leading to a lower ability to trigger rapid insulin release via the calcium dependent route [50].

Careful regulation of calcium is key for mitochondrial activity. Mitochondria are organelles that store calcium, however, not at a significant concentration to effect cytosolic concentrations [51]. The relationship of mitochondria with calcium is two-fold: ATP production and calcium-trafficking or redistribution. Calcium uptake by mitochondria enhances their ATP production potential; however, a fine balance must be struck, as calcium is required, but too much calcium induces apoptosis. In-order to manage this, calcium efflux must be effective to avoid overload [52]. Mitochondrial calcium uptake is mediated by the calcium uniporter channel complexes (MCUC), while mitochondrial calcium extrusion is facilitated by the sodium/calcium exchanger (NCLX). Both the MCUC and NCLX are electrogenically driven by the high ΔΨm [52,53]. Hyperinsulinaemia (HI) increases mtROS production via ceramide synthesis, and NAD+ depletion from concomitant elevated glucose-metabolism, leading to decreased counter-ROS management. Excess ROS generation increases UCP2 in beta cell mitochondria, resulting in a decrease in the ΔΨm [54]. This impairs the mitochondrial MCUC and NCLX dependent uptake and the redistribution of calcium that is required for maximal ATP synthesis [51,55]. The second role of mitochondrial calcium homeostasis is focused on redistribution efforts [53,56].

Mitochondria facilitate the trafficking of cytosolic calcium uptake into the endoplasmic and sarcoplasmic reticulae via Ca^2+^ ATPase (SERCA) pumps [57,58]. The beta cell endoplasmic reticulum (ER) regulates cytosolic calcium partitioning, while ATP dependent SERCA pumps dominate in mediating calcium exocytosis, in-order to “re-set” PM calcium levels. These mechanisms enable the cycle of the calcium-mediated exocytosis of insulin granules packaged in vesicles, docked along the inner PM, to repeat [59]. Roughly 20% of the beta cell sub-plasma membrane is in close proximity with mitochondria, exerting a strong calcium buffering effect [22]. Mitochondrial calcium uptake facilitates the signal:response ratio sensitivity in PM depolarisation and increases the cytosolic calcium concentration [60]. A reduction in the mitochondrial calcium uptake via reduced MCUC activity, leads to an increase in calcium within the PM sub-membrane compartment upon depolarisation [29,55]. This attenuates any increase in cytoplasmic calcium and results in a net reduction in rapid insulin exocytosis. An increase in ROS increases UCP2 expression, which lowers the ΔΨm, consequently reducing calcium uptake and efflux [20,49,50,52]. As a result, this not only decreases rapid ATP synthesis potential, but also impairs calcium trafficking to the ER [61]. As a consequence, dysregulated cytosolic calcium levels may reduce/impair the docking “ready–set–go” positions of the exocytosis mediating SNAP/SNARE proteins [62,63,64]. This impairment results in the disabling of a rapid response from the exocytosis machinery to the required extracellular calcium influx along the PM, of the much-needed steep calcium concentration gradient that is sensitive to the signal:response ratio to elicit rapid insulin release. In short, when the concentration gradient is not steep, the signal is not strong, resulting in a poor response.

Excessive ROS levels, along with chronic insulin signalling, increases the ratio of dynamin-related protein 1 (Drp1) to mitofusin-2 proteins (Mfn2) [65,66]. Drp1 mediates mitochondria fission, while Mfn2 mediates mitochondrial fusion and is required for ER association [51]. When there is an increase in Drp1 relative to Mfn2, there is a net increase in mitochondrial fission [51,66]. This results in a decrease in ER association and OxPhos capacity, and an increase in mtROS production [66,67]. Consequently there is an increase in ER stress, a reduction in ER mediated calcium homeostasis, and a reduction in mitochondrial (mt) OxPhos that is required in order to generate the ATP surge needed for first phase insulin exocytosis [58,68]. It is, therefore, clear that the health of beta cell mitochondria are essential for a functional first phase response to a glucose bolus [54,69,70].

## 4. Insulin Secretion in the Insulin Resistant/Hyperinsulinaemic State

Having established the processes in the healthy state, the hyperinsulinaemic individual can be considered. However, a number of factors first need to be addressed in the research literature on T2DM and pancreatic beta cells. A large majority of the literature states that in T2DM, there is a significant loss of beta cell mass, and this, consequently, results in insulin insufficiency [71,72]. However, this stage of T2DM is the far end of the condition, where the pathology is entering into the final stages of T2DM-induced pseudo-T1DM, as a result of beta cell “exhaustion”, failure and/or increased apoptosis [72]. However, in many people, this is a relative deficiency [73], not an absolute, as examination of the Kraft dataset shows they still produce more insulin than the normoglycaemic/normoinsulinaemia population [36].

**Figure 3:**
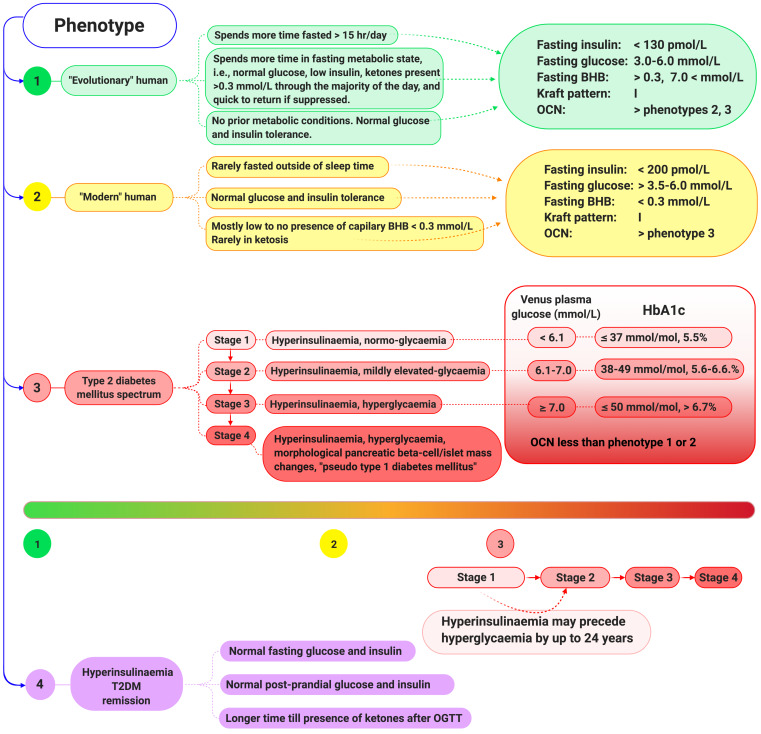
Classification of metabolic phenotypes. Beta-Hydroxybutyrate (BHB), haemoglobin A1c (HbA1c), oral glucose tolerance test (OGTT), osteocalcin (OCN), type 2 diabetes mellitus (T2DM).

A large phase of the pathogenesis of T2DM is the silent normo-glycaemia hyperinsulinaemia phase, often termed (pre-)pre-diabetes. In reality, this phase would be best termed stage-1 T2DM and mildly elevated glycaemia, currently termed pre-diabetes, stage-2 T2DM (Figure 3). Waiting to see hyperglycaemia (HG) (stage-2/3), in-order to diagnose T2DM, is already deep into pathology progression, where chronic excess insulin levels are no longer able to mask the problem. Obese individuals are more likely to have stage-1/2 T2 diabetes and have a higher risk of developing HG-T2DM (phenotype-3 stage 3) than non-obese individuals. An abnormally high percentage of islet tissue, and increased beta cell mass has been found in the pancreas of obese individuals in comparison to lean subjects [74,75]. Given the scale and changes in phenotype along the trajectory pathogenesis of T2DM, it is vital that distinctions are made between the stages. Furthermore, that investigations in biological samples and participants belonging to different pathology stage categories, are not pooled. This is to avoid cancelling out effects/observations between two or more stages. For example, stage-1/2 normo-glycaemia HI T2DM with increased beta cell mass, pooled with stage-3/4 HG-HI T2DM with decreased beta cell mass [73]. In this example, the net pooling results in a cancelling out of any signal. This can lead to the forming of incorrect premises that would contribute to a misinterpretation of results and, in addition, potentially cause the development of in vitro and animal models that do not truly represent the full scope of the disease. Again, this, consequently, increases the risk of producing results that are correct for the experiment only, which is based on a flawed premise, in turn, sending the researcher on a merry-go-round.

## 5. An Alternative Hypothesis: Insulin’s Main Role Is to Regulate Beta-Hydroxybutyrate Synthesis

Let us assume the natural human state is to be in a hunter–gatherer pattern, the equivalent is hypothesised to be found in the early European exploration of the traditional Inuit and the Hadza examples. These societies are characterised by predominantly fasted—metabolic phenotype 1 (Figure 3), often only consuming one meal a day, potentially not having food every day, and most meals are low in digestible carbohydrates [76,77,78]. In this context, blood glucose levels infrequently rise above 6 mmol/L, only with occasional access to honey or fruit, or a meal containing a high glycogen content, such as liver. Alternatively, blood glucose may surge in response to an acute stress response. Aside from these contexts, blood glucose levels remain relatively constant and may even dip to levels that conventional medical wisdom considers puts the individual at risk of a hypoglycaemic coma [79]. However, it has been demonstrated that humans in nutritional ketosis are able to function comparably, even optimally, when plasma glucose levels are below the standard reference ranges, due to the elevated presence of the ketone body beta-hydroxybutyrate (BHB) [80]. The fasted state, or carbohydrate restriction, induces the metabolic phenotype of ketosis, where plasma insulin is low, glucose is normal to low, and BHB is detectable above 0.5 mmol/L [43]. Just as hyperglycaemia may become pathological, hyperketonaemia may also become pathological, especially when BHB levels exceed 25 mmol/L. Within humans, diabetic ketoacidosis (DKA) pathology is when there are elevated ketones, with hyperglycaemia and decreased bicarbonate levels, resulting in a decrease in blood pH [81,82,83]. Other common symptoms experienced with DKA include nausea, vomiting, and gastrointestinal symptoms including abdominal pain [84]. Insulin regulates hepatocyte BHB synthesis, therefore hyperinsulinaemic individuals are at a very low risk of developing DKA, unless they are on sodium-glucose co-transporter-2 (SGLT2) inhibitors and simultaneously embark on carbohydrate restriction without adjusting medications [43,85].

If the natural state of humans is to spend more time in the metabolically fasted phenotype of ketosis (phenotype 1), it is plausible that the role of basal pulsatile insulin secretion is to regulate BHB synthesis. Cells that are wholly or substantially glucose dependent do not require insulin to take up glucose [86,87]. In the fasted state, blood glucose is provided from the liver, either from the glycogen stored from a rare glucose loaded meal or, more likely, from gluconeogenesis that both replenishes hepatic glycogen stores as well as providing extra-hepatic tissue needs [88,89]. In this context, hepatocytes are metabolising fatty acids for their own energy provision and in the process synthesise BHB [90]. Hepatocytes are unable to use BHB for fuel [91], instead the BHB is released into the bloodstream, to provide energy and act as a signalling molecule to extra-hepatic tissues, such as the brain, heart, and muscular-skeletal system [46,92]. In the absence of insulin production, BHB synthesis would continue unabated, as seen in type 1 diabetes mellitus (T1DM). However, a small amount of insulin is able to inhibit ketogenesis [93]. The signal for insulin release is required though, this then begs the question, what provides the signal?

It is first important to understand that when humans are in ketosis as their de-facto state, the body’s supply of glucose is dependent on hepatic synthesis and provision [30,89]. It is essential that the liver does not respond to insulin’s effects on glucose output, even for one meal. This is because, hypothetically, one high carbohydrate meal that would induce a high insulin output, could shut-down hepatic glycogenolysis and gluconeogenesis. This insulin signalling effect may overshoot in duration, resulting in depriving glucose dependent cells of hepatic glucose, fatty acids, and BHB, which are also regulated by insulin [89]. The liver becomes physiologically “IR”, which is really a state of adaptive homeostasis. In reality, the liver is not uniformly IR, as it can be seen, a glucose bolus in keto-adapted individuals rapidly curtails BHB synthesis, whilst not inhibiting hepatic glucose output. The hepatic “IR” is selective [94,95].

**Figure 4:**
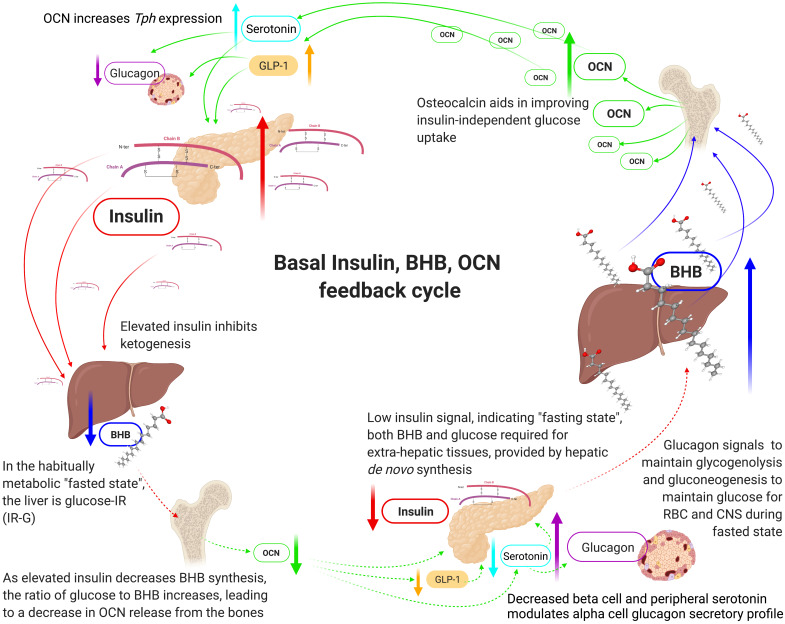
Proposed basal insulin, beta-hydroxybutyrate, osteocalcin feedback cycle in phenotype-1 individuals. Beta-hydroxybutyrate (BHB), central nervous system (CNS), glucagon-like peptide-1 (GLP-1), insulin resistance (IR), glucose-insulin resistance (IR-G), osteocalcin (OCN), red blood cells (RBC), tryptophan hydroxylase (*Tph*).

Individuals in habitual ketosis have significantly lower glucagon and insulin levels than hyperinsulinaemia T2DM patients [31,43,80,96]. Hepatic glucose output is both regulated by glucagon and is likely due to the continual “draw-down” of plasma glucose by extra-hepatic tissues [31,97]. As BHB rises and glucose levels are restricted, this stimulates an increase in osteocalcin (OCN) synthesis and release from the bone by osteoblasts, osteocytes, and osteoclasts [98,99,100]. OCN significantly increases glucose uptake independent of insulin and, in the fasted state, likely functions as the glucose uptake regulator, rather than insulin [101,102]. However, OCN potentiates insulin’s glucose uptake effect, therefore requiring less insulin, which effectively improves insulin sensitivity with regard to glucose uptake [103]. OCN also induces glucagon-like peptide 1 (GLP-1) synthesis. Both OCN and GLP1 signal beta cells to increase insulin synthesis and release [101,104,105]. The resulting effect is that elevated BHB and glucose restriction drives OCN synthesis and release, and OCN increases GLP-1 [105]. Together, OCN and GLP-1 increase insulin secretion [106,107], resulting in downregulating BHB synthesis, which, in turn, down regulates OCN release from the bone, which removes the signal for insulin secretion, and, therefore, insulin levels decrease. This feedback loop effectively regulates BHB synthesis and glucose homeostasis (Figure 4). It is also intriguing that the half-life of OCN and GLP-1 are both 5 min, and the pulsatile pattern of insulin secretion is 4 to 15 min [20,108,109]. The combined effects of OCN and GLP-1 may enhance the signal for insulin secretion to potentially match or synergise in generating the following feedback cycle: BHB increase → OCN increase (+GLP-1) → insulin release → BHB decrease → OCN decrease → insulin decrease → BHB increase. Furthermore, BHB, together with lactate or low levels of glucose, potentiates the strength of the signal for insulin release, indicating that BHB directly, although not independently, stimulates an insulin response [110].

It would be remiss to not indicate the significant role of the pancreatic alpha cells in glucose and insulin homeostasis. A higher insulin level should predict a lower fasting plasma glucose. However, as we see, fasting glucose is elevated in T2DM, as well as higher glucagon and insulin [36,96]. The question is why would the alpha cells be secreting more glucagon in a higher glucose background? An explanation maybe found in OCNs role in regulating tryptophan hydroxylase (*Tph*) gene expression, the rate limiting enzyme for serotonin (5-hydroxytryptamine, 5-HT) synthesis, an alpha cell glucagon secretory profile modulator [111,112].

Through a series of elegant experiments, Almaça et al.,(2017) demonstrated in vivo and ex vivo that alpha cell secretion of glucagon is modulated by serotonin signalling via the 5-HT1F receptor, causing rapid inhibition of adenylate cyclase, resulting in a decrease in intracellular second messenger cyclic adenosine monophosphate (cAMP). Furthermore, beta cells from people with normal glucose tolerance, phenotypes 1 and 2, produce and secrete serotonin, and alpha cells respond to this serotonin, leading to the modulation of glucagon secretion under different plasma glucose conditions. Almaça et al. showed that when islet serotonin levels are manipulated (serotonin is depleted or inhibited), alpha cells lose their ability to respond in concordance to surrounding glucose levels. This affects glucose homeostasis, and likely plays a negative role in propagating pernicious increases in fasting basal insulin levels, a pattern seen in the progression toward hyperinsulinaemia phenotype-3 stages 1 and 2, and overt T2DM phenotype-3 stage 3.

Serotonin is a strong paracrine regulator of the secretory profile of alpha cells [112]. Higher levels of serotonin decrease alpha cell glucagon secretion when plasma glucose levels are high and also reduces the amount of glucagon secretion at very low glucose levels [112]. This may explain why phenotype-1 (longstanding metabolically flexible habitual ketosis) and phenotype-2 individuals would have lower glucagon and insulin levels, while maintaining lower glucose levels than found in those with hyperinsulinaemia phenotype-3 [30,96].

GLP-1 receptor activation, in vivo, increases serotonin synthesis [113]. Furthermore, serotonin has been found to be synthesised and stored with insulin inside the beta cell insulin secretory beta-granules, and co-released upon glucose stimulation [114]. Beta cell serotonin secretion shares similarity to the secretory pattern of insulin, having a glucose-dependent and pulsatile pattern [112]. GLP-1 is also able to directly suppress glucagon secretion via inhibiting alpha cell P/Q-type-voltage-gated Ca^2+^ channels [26,115].

OCN increases serotonin and GLP-1 synthesis, both of which modulate the alpha cell glucagon secretory profile. OCN levels are significantly lower in insulin resistant and T2DM individuals [99,116,117,118]. Glucose restriction and lower insulin levels enhance osteoblastogenesis and osteocytogenesis, and OCN synthesis and release. This provides a plausible metabolic and endocrine regulatory feedback cycle (Figure 4), in maintaining glucose homeostasis, whilst also maintaining lower glucagon and insulin levels in phenotype-1 and -2 individuals relative to phenotype-3 [100,119].

Consider an alternative basic premise; humans evolutionarily spent more time in a metabolically fasted state of ketosis than current modern-day humans (phenotype 2 and 3). Then, plausibly, the role of insulin is to regulate BHB synthesis, while the role of bone regulates glucose uptake sensitivity via OCN. OCN then regulates fasting glucose levels via regulating the alpha cell glucagon secretory profile and BHB synthesis via regulating insulin release. An acute “fight or flight” stress response, such as running from danger or running for hunting, would induce a rapid glucocorticoid stimulated hepatic release of glucose that would then signal for a rapid insulin secretion response [120]. The liver is “IR” toward glucose output, to ensure that the glucose may be provided for increased muscle uptake, increased red blood cell (RBC) use for oxygen transport, and to increase the clotting ability in the case of potential physical harm that could cause haemorrhage and subsequent life-threatening hypovolemia.

The acute hyperinsulinaemia that accompanies the acute hyperglycaemia, inhibits anti-coagulation processes via upregulating plasminogen activator type 1 (PAI-1), and thus upregulates blood coagulability [121,122]. It, therefore, stands to reason that, in the fasted state, hepatic glucose output mechanisms would adapt to not respond and, thus, be inhibited by a rapid surge in insulin secretion, because if it did, then the “glucose tap” would be turned off. This would result in sudden life-threatening deprivation of glucose for the RBCs and certain parts of the central nervous system (CNS), which must receive glucose.

It is equally “essential” that the liver becomes IR in the context of hyperinsulinaemia as well. When a significant enough portion of time is spent in the fed state, which includes carbohydrate consumption, rapid insulin secretion is induced; over time, this down regulates the expression of BHB synthesis enzymes [90,93,123,124]. As a result, the return to ketogenesis does not occur within 3 to 5 hours post prandial. Furthermore, chronic hyperinsulinaemia impairs hepatic and extra-hepatic beta-oxidation, driving a greater extra-hepatic reliance on glucose for fuel [125]. Consequently, glucose becomes the essential fuel for the system, a system that does not easily “switch gears” to using fatty acids nor BHB, which is not readily available. Again, in this circumstance, the liver must continue to release glucose even when there is an external influx of exogenous glucose causing a rapid insulin release [94]. It may be the case that the down regulation of the rapid insulin release may not be wholly pathological if one considers it as a means to reduce any excess insulin signal on the liver to inhibit glycogenolysis. However, this is hypothetical and it is more likely that mitochondrial damage results in the pathological changes that impair the first phase rapid insulin response after a glucose bolus in T2DM patients, and this, in turn, contributes to a pathological feedforward progression of increases in fasting basal insulin levels [29,50,55,126].

The first phase rapid insulin response is impaired in hyperglycaemic T2DM patients [5,36], and may be a significant T2DM risk marker for hyperinsulinaemic/normoglycaemic people (phenotype 3, stage 1) [127]. There is a danger in thinking the solution is to find a way to stimulate the rapid exocytosis of a high concentration of insulin to rapidly lower plasma glucose levels. Although, that would result in what would appear to be, better glucose homeostasis. However, it must be remembered that these patients are also insulin resistant and have hyperinsulinaemia. Finding a way to help individuals with T2DM to activate the rapid first phase response, i.e., increased insulin, would likely only potentiate the insulin resistance and further increase their hyperinsulinaemia, which is present for a period of time preceding hyperglycaemia. The better strategy is to understand what causes the damage to the first phase response. Once this is understood, then the logical action is to remove the detrimental upstream causal stimulus, shown successfully by a variety of different carbohydrate and/or calorie restricted processes [78,128,129,130,131].

The current paradigm for insulin secretion regulation, for both basal and bolus, is that glucose is the primary stimulus. Our hypothesis challenges this and proposes a new paradigm: that bolus insulin secretion is regulated by glucose stimulus, but basal insulin secretion is regulated by OCN. As people spend more time in a higher glycaemic state, for example decreased fasting, constantly post prandial, this increases the frequency of the requirements of the bolus secretion dominance, with plasma glucose being above 6 mmol/L, which suppresses the role of basal insulin secretion. As a result, reduced OCN-regulated signalling diminishes serotonin regulation on glucagon output and, consequently, fasting glucose rises again, potentiating bolus insulin release.

This proposal, regarding OCN regulating basal insulin release, needs to be thoroughly tested, for example, in phenotype 1 people, who could be proxied by people in habitual ketosis with no prior metabolic health dysregulation. Following an overnight fast, plasma samples for glucose, insulin, BHB, OCN, GLP1, glucagon, and serotonin are collected at least every 5 min for 60 min, to establish basal state insulin secretion. These people are then given a glucose bolus, to establish a phenotype 1 OGTT metabolic profile.

Further investigations into insulin responses upon oral glucose tolerance tests (OGTT), in individuals with phenotype 1, will likely demonstrate healthy first phase rapid insulin secretion responses. These individuals have no prior metabolic health conditions and maintain a longstanding habitual metabolic fasting-mimicking phenotype. In response to an OGTT, their hepatic glucose output will not decrease; however, BHB synthesis will be decreased/inhibited. The return of ketogenesis then marks the decrease in bolus stimulated insulin signalling, while exogenous and endogenous glucose are both put away, out of the bloodstream in a timely manner, somewhat akin to a healthy transient acute stress response. While this may turn out to be true in acute infrequent trials, chronic glucose tolerance tests, in the form of “three square meals a day”, likely induce the pathological changes that evolve into the chronic diseases associated with ageing.

## 6. Clinical Implications

Clinically, this new paradigm suggests that there needs to be an expansion in the concept of good metabolic health to include the presence of BHB. While ketone esters are available as health supplements, future research needs to consider whether there is a difference in clinical outcomes between endogenous and exogenous BHB. Furthermore, we need a better understanding of the metabolic profiles of people representative of the phenotypes, as described above. For example, people with phenotype 1 are likely to have a greater glucose sparing effect, they may have a heightened glycaemic response following a glucose bolus, compared to people with phenotype 2 or 3. Using multiple metabolic markers, especially the combination of glucose, insulin, BHB, and, ideally, OCN, may provide a better understanding of metabolic or endocrine health. Understanding basal insulin regulation enables earlier detection in changes that are associated with progression in the pathological development of hyperinsulinaemia conditions. With this understanding, it becomes clear that tight glycaemic control via medications that directly increase insulin levels, especially the sulphonylureas or exogenous insulin, only further potentiates hyperinsulinaemia and subsequent pathologies [132]. Lifestyle management, especially carbohydrate restriction, [43,128,130] with adequate individualised support should be considered first line treatment for hyperinsulinaemia, with or without hyperglycaemia, as it offers a more effective method in improving glucose homeostasis whilst also decreasing excess insulin exposure.

## 7. Conclusions

Once the metabolic phenotype is established, this would enable better selection criteria and grouping for participant/tissue sampling/cell culture-media experimentation. Thus, it would enable the avoidance of pooling participants/patients results that lead to signal cancelling effects. This will then aid in the pursuit of resolving the regulation of basal insulin secretion. Understanding the individual phenotype and stage allows for better patient understanding and compliance with clinical nutritional management.

## Data Availability

Data sharing not applicable as no datasets were generated and/or analysed for this study.
